# Categorising drivers of curriculum renewal in entry‐to‐practice health professional education: A scoping review

**DOI:** 10.1111/medu.15614

**Published:** 2025-02-19

**Authors:** David L. Kok, Mary Xu, Stephen Trumble, Kristie Matthews, Caroline Wright

**Affiliations:** ^1^ School of Primary and Allied Health Care Monash University Melbourne Australia; ^2^ Department of Radiation Oncology Peter MacCallum Cancer Centre Melbourne Australia; ^3^ Faculty of Medicine, Dentistry and Health Sciences University of Melbourne Melbourne Australia; ^4^ Monash Centre for Scholarship in Health Education Monash University Melbourne Australia

## Abstract

**Introduction:**

Renewing a health professional education (HPE) curriculum is a significant undertaking, requiring substantial effort and resourcing. However, the factor(s) that should initiate a curriculum renewal remain contentious, and the methods to best assess them are unclear. To begin answering these questions, a comprehensive view of the scope of potential renewal drivers is needed, thus enabling structured inquiry into these (and their interactions) while ensuring no driver categories are being inadvertently overlooked. It would similarly serve as an important checklist for individual institutions undergoing renewal. However, no such list has ever been formulated in a data‐driven way. Thus, this scoping review aimed to document the full range of real‐world drivers of HPE curricular renewal and create an overarching categorisation of these.

**Methods:**

A systematic search was conducted of the Embase, Medline/PubMed, Global health and CINAHL databases for studies published between 2013 and 2023 describing a completed single‐institution curriculum renewal of an entry‐to‐practice HPE degree. Following independent screening, articles were reviewed and ‘extent of renewal’, ‘renewal characteristics’ and the ‘initiating driver(s)’ of renewal were extracted. Descriptive statistics were generated, and qualitative content analysis performed to generate a categorisation of renewal‐initiating drivers.

**Results:**

The search identified 1408 potential manuscripts, and 239 publications describing 247 renewals were analysed. Forty percent were whole‐of‐degree renewals. Institutions from all continents were represented. Seventy‐seven initiating drivers were identified and grouped into 10 categories: ‘Regulatory reasons’, ‘Pedagogical improvements’, ‘Community/patient needs’, ‘Learner experience/satisfaction’, ‘Exceptional events’, ‘Learner outcomes’, ‘Industry benchmarking/reputation’, ‘Practical/business factors’, ‘Knowledge/skill issues’ and ‘Periodic renewals’. ‘Regulatory reasons’ were the most common reason for whole‐of‐degree renewals and ‘knowledge/skill issues’ for part‐of‐degree renewals.

**Conclusions:**

HPE curricula renewals have a wide range of initiating drivers. The proposed categorisation of these should serve as a useful scaffold for institutional curricular review processes and also for directing future scholarly inquiry into curricular renewal.

## Abstract 2

Health professional educators are constantly renewing curricula, but why? This review proposes a comprehensive framework to scaffold and assess renewal processes.

## 1 INTRODUCTION

Health professional education (HPE) curricula are constantly undergoing renewal to align with contemporary best practice.^1^, ^2^, ^3^, ^4^, ^5^, ^6^ Renewals are sizeable undertakings and require significant resourcing in terms of time, personnel and finance.^2^, ^7^ In addition, while curricular reform has many benefits, it can also result in unforeseen negative consequences including affecting student well‐being^8^, ^9^ and rates of staff burnout.^10^, ^11^ These effects may be further compounded in settings where there is limited baseline health and education resourcing.^12^, ^13^ Therefore, ensuring HPE curricula renewals are being judiciously initiated for worthwhile reasons is vital.^2^, ^14^, ^15^, ^16^ Or, as eloquently stated by Bland, ‘It may seem self‐evident that an organization would not embark on an innovative path without first identifying a bona fide ‘need’ for change’.^7^

As self‐evident as this may be, numerous members of the HPE community have raised concerns that this does not always seem to be the case.^2^, ^17^, ^18^, ^19^, ^20^ The root cause of this tension is not because educators are wilfully initiating renewals for injudicious reasons, but rather because there is minimal agreement on the appropriate factor(s) that should drive a curricular renewal and how they can be objectively assessed.

If the HPE community is to even begin to answer these questions, it is first important to know the full scope of possible reasons for initiating a curricular renewal. Currently, there is no accepted comprehensive classification system of renewal drivers, making navigation of this complex space difficult. Without such a framework, renewals often evolve markedly between when they are initiated and completed^21^, ^22^ as additional unanticipated ‘needs’ are unearthed during the process. While lists of potential drivers of curricula change do exist in the HPE literature,^1^, ^23^, ^24^, ^25^ these are based on expert opinion and compiled in a narrative rather than systematic way. To our knowledge, the only systematic, data‐driven synthesis on an analogous topic in HPE is that by Christakis,^26^ published in 1995, which summarised the recommended reasons for reform of US medical schools based on 19 health association reports. The utility of these findings is limited due to the age of the publication, reliance on recommendations (rather than actual institutional practice) and unclear relevance outside the US medical education setting.

Curriculum renewal is also a topic of interest in the broader higher education literature. Again, a number of lists of potential drivers of curriculum change have been proffered,^27^, ^28^, ^29^, ^30^ including a notable white paper by the International Bureau of Education.^27^ However, these retain similar limitations to the HPE‐specific publications, namely, being narrative in nature and lacking a systematic, data‐driven method of compilation.

Therefore, establishing a comprehensive, data‐derived, categorisation system would significantly facilitate structured academic inquiry into curriculum renewal drivers and their interactions.^20^, ^31^ In addition, it would serve as a useful reference for individual institutions considering a renewal to ensure they have considered all potential needs from the outset, ensuring no driver categories are being inadvertently overlooked.^31^

Thus, this study was conceived to (a) compile a comprehensive view of the full spectrum of real‐life drivers of international HPE curricula renewal, (b) generate a proposed categorisation of these drivers to scaffold both individual institutional curricular renewals and future academic inquiry and (c) provide initial insights into the current utilisation of each identified driver category.

The primary research questions for this scoping review were as follows:

(1.a) What are the initiating drivers of entry‐to‐practice HPE curricula renewals?
(1.b) What are the broad categories that these drivers may be grouped into?

The secondary research question for this study was:

(2) What is the frequency and distribution of utilisation of each driver category?

## 2 METHODS

We employed a structured scoping review methodology following the framework described by Arksey and O'Malley^32^ and Levac et al.^33^ The extraction, analysis and reporting of findings are aligned to the recommendations of Pollock et al.^34^

### 2.1 Eligibility criteria

This review was inclusive of published studies related to entry‐to‐practice health professions programmes. We chose to specifically focus on clinical entry‐to‐practice qualifications due to their core role in HPE and for feasibility reasons. To ensure that our findings represented actual drivers of curricular renewal (i.e. not just theorised drivers), only first hand published accounts or evaluations of a successfully completed single‐institution curricular renewal were included. In addition, the publication was required to have a clear statement articulating the reason(s) the renewal had been initiated (i.e. an explicit ‘cause and effect statement’ with a specific ‘cause’ leading directly to renewal initiation. Examples of this are included in the result section).

A 10‐year publication window was chosen, from 2013 to 2023. A Population/Concept/Context^34^ summary for the scoping review is as follows:

**Population:** Any health professional discipline, worldwide

**Concept:** Initiating drivers of curricular renewal in Health Professional Education

**Context:** Entry to Practice Health Professional Education degrees, published 2013–2023

For the purposes of this study, a ‘renewal’ was defined as any update/change to an existing HPE curriculum, regardless of extent. However, extent was quantified in our extraction to stratify analysis—these were divided into ‘whole‐of‐degree’ renewals (where the entirety of a HPE degree programme was revised) and ‘part‐of‐degree’ renewals (where only a portion of a degree programme was revised, e.g. a module or specific year of study). Brand new degrees were excluded, as these were not renewals. However, addition of new subjects/topics within a degree were included as these are a change to a portion of an existing degree curriculum.

### 2.2 Information sources and search strategy

In October 2023, the Embase, Medline/PubMed, Global health and CINAHL databases were searched for articles published in English between January 2013 and September 2023.

The search strategy was developed by the research team in consultation with a librarian who had extensive expertise in health science, education and database terminology. Three main concepts were utilised: ‘all health professions’, ‘entry to practice qualifications’ and ‘curriculum renewal’. Core keywords, stemwords and synonyms were identified for each concept. The keyword variations were cumulatively grouped using the (OR) Boolean connector, and the main concepts were linked using the (AND) Boolean connector. Further details on the core concepts and search strategy are included in Appendix S1.

### 2.3 Study selection

Search results were imported into Covidence (Covidence systematic review software, Veritas Health Innovation) and duplicates identified and removed.

All five named authors of this manuscript participated in the screening process, with a minimum of two reviewers independently screening each title and abstract to determine eligibility for full‐text review. Conflicts were resolved by discussion, to reach a consensus. The full‐texts of all identified studies were then reviewed by the first author and a second investigator who reviewed a randomly selected sample comprising 10% of the studies (as per^34^, ^45^, ^46^). When compared, concordance was very high (98.7%), and thus further review was deemed unnecessary.

Of note, publications that only described a proposed or recommended curricula renewal (i.e. that had not yet been implemented) were excluded as, for validity reasons, we only wanted drivers that had definitively resulted in curricular renewal. For similar reasons, commentaries/reviews that gave opinions on potential reasons for curricular renewal were not included, although their references were manually searched and if these yielded relevant references that fit inclusion criteria they were included for review.

### 2.4 Data charting and analysis

A data charting tool and database were developed using REDCap electronic data capture tools.^47^ Data fields included the authors, year of publication, year renewal implemented, publication type, relevant clinical discipline(s), country, extent of renewal, renewal specifics, driver of renewal, driving group and if an evaluation process were used to identify the driver.

For the first 100 publications, the entire driver statement(s) was copied verbatim from the source publication into the charting tool. At this point, a set of codes was generated by DK that represented each individual driver concept encountered in the data. These codes were discussed and agreed upon with KM and CW and added to the charting tool. All manuscripts were then charted (including both the verbatim driver and coding) by DK. Once this first round of charting was completed, four additional codes were added to the charting tool representing drivers that had not been encountered in the initial 100 publications. A second investigator (MX) then independently charted a random sample representing 10% of the publications. Data concordance was again extremely high (97.2%), and the need for further review deemed unnecessary.

Conventional content analysis (CCA) was utilised to inductively construct a categorisation system for the renewal drivers.^48^, ^49^ CCA was chosen as its strengths aligned well with our desire to construct a de novo categorisation that was grounded in the data and not based upon any previously suggested systems.^49^ An initial set of subcategories, categories and definition of the categories was drafted by DK then discussed and refined with all co‐authors. Consensus was reached between all authors on the final categories and their definitions. These categories were then applied to the dataset in REDCap (with records retained of all intermediate categorisation steps).

Once finalised, summary characteristics of the identified studies were generated both in tabular and visual format in SPSS (IBM SPSS Statistics for Windows, Version 29.0.2.0, IBM Corp). Quantitative content analysis was also conducted to describe the frequency and percentage of each renewal driver category.

## 3 RESULTS

### 3.1 Overview

**FIGURE 1:**
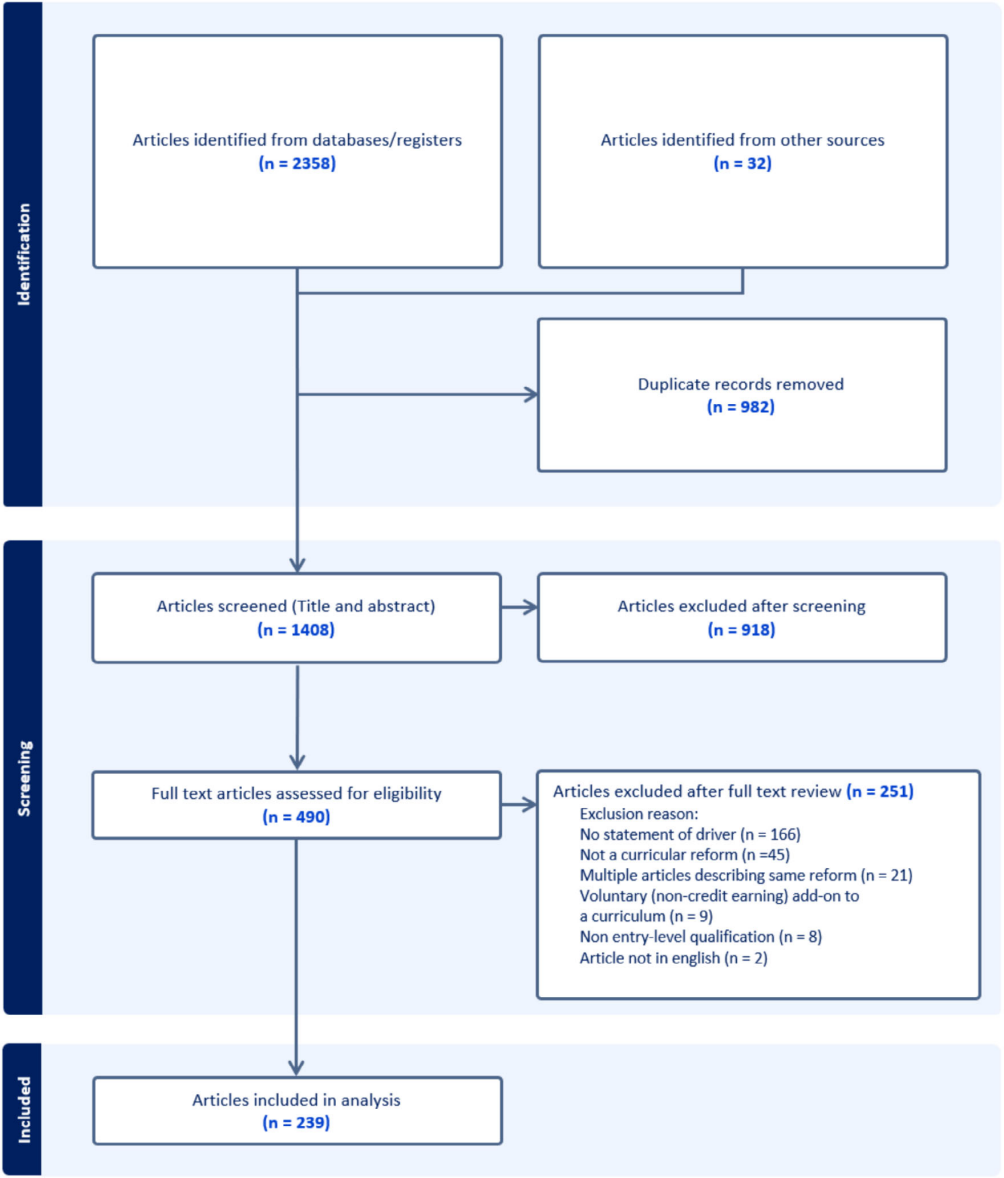
PRISMA diagram. [Color figure can be viewed at wileyonlinelibrary.com]

Of the 2390 articles identified from our search strategy, 982 were identified as duplicates and removed. Title and abstract screening were thus performed of 1408 articles. Following full‐text review, ultimately 239 articles were included in the analysis, which described 247 renewals in total (NB. some articles described more than one renewal). See Figure 1. A full list of included studies is in Appendix S2.

### 3.2 Characteristics of the studies

The curricula renewals were conducted in a broad range of countries, with representation across all continents. North America was the predominant region (*n* = 132, 53.4%), followed by Europe (*n* = 48, 19.4%) and Asia (*n* = 37, 15.0%). Similarly, a broad selection of health professional programmes were represented in the collated studies, with medicine accounting for the predominant proportion (*n* = 156, 63.2%), followed by nursing (*n* = 43, 17.4%), pharmacy (*n* = 26, 10.5%) and dentistry (*n* = 18, 7.3%). See Table 1 and Figure S1a.

**TABLE 1:** Characteristics of the studies.

		No. of renewals (*n* = 247)	%
Country of origin	USA	119	48.2%
	Canada	13	5.3%
	Australia	12	4.9%
	Netherlands	11	4.5%
	Germany	10	4.0%
	India	7	2.8%
	Great Britain	6	2.4%
	Brazil	5	2.0%
	Ireland, Iran, Switzerland and Saudi Arabia	4	1.6%
	South Africa and Taiwan	3	1.2%
	China, Ecuador, Iraq, Malawi, Qatar, Singapore and Vietnam	2	0.8%
	Austria, Bahrain, Belgium, Colombia, Croatia, Curacao, Finland, Greece, Hong Kong, Israel, Italy, Japan, Korea, Kuwait, Lebanon, Mexico, New Zealand, Norway, Pakistan, Portugal, Serbia, Slovakia, Spain, Sri Lanka, Tanzania, United Arab Emirates, Vienna and Zimbabwe	1	0.4%
Health professional discipline (more than one may apply)	Medicine	156	63.2%
	Nursing	43	17.4%
	Pharmacy	26	10.5%
	Dentist/dental hygienist	18	7.3%
	Physiotherapy/physical therapy/exercise physiology	5	2.0%
	Dietetics	3	1.2%
	Radiotherapy/radiography	3	1.2%
	Social work	2	0.8%
	Psychology	1	0.4%
	Speech pathology/therapy	1	0.4%
	Paramedic	1	0.4%
	Occupational therapy	1	0.4%
	Midwifery	1	0.4%

The renewals were implemented across a timespan from 1995 to 2023, following a standard distribution (median = 2014, std dev = 5.2). A notable anomaly was in 2020 when there was a significant spike in renewals correlating with the Covid‐19 pandemic (Figure S1b).

In terms of renewal size, 99 of the 247 renewals (40.0%) were ‘whole‐of‐degree’ renewals and 60.0% ‘part‐of‐degree’. Renewals that were smaller than an individual subject were the next largest subcategory comprising 25.1% of the total number (62/247) (Figure S1c).

For whole‐of‐degree renewals, a small proportion were transitions to online delivery (17/99, 17.2%), and the vast majority involved major pedagogical change (79/99, 79.8%). Many part‐of‐degree renewals also had significant changes in pedagogy (53/148, 42.6%) and an even greater proportion had new or changed topic/subject matter (90/148, 60.8%) (Table S2).

### 3.3 Initiating drivers and their categorisation

From the 247 renewals, 528 drivers were identified, in total, for an average of 2.15 driving reasons per renewal (range 1–12, mode = 1).

Identical drivers were matched, resulting in 77 individual drivers identified. **A complete list of these drivers is in** Appendix S3.

Through CCA, 10 categories of initiating drivers were formulated. The definitions, example driver statements and example driver codes are listed in Table 2.

**TABLE 2:** Proposed definitions and categories of initiating drivers, including examples.

**Category 1: Knowledge/skill issues**	**Definition**	Reasons pertaining to the representation of knowledge or skill within a curriculum, including its presence/absence, currency or relative weighting
	**Example driver codes**	Topic underrepresented; topic overrepresented; topic requires updating to be more contemporary (including removal of outdated knowledge)
	**Example driver statement**	‘gap analysis identified underdeveloped competencies in safety knowledge and skills measurable in both clinical and classroom courses […] In response to identified gaps in safety competencies, new objectives were developed, leveled for clinical and classroom courses and introduced throughout the four‐year program’. Weatherford, 2015^35^
**Category 2: Exceptional events**	**Definition**	Reasons pertaining to exceptional events of an external nature
	**Example driver codes**	Covid‐19; War
	**Example driver statement**	‘This paper describes the redesign, due to the Covid‐19 pandemic, of classroom‐based patient‐facing activities into an online format’. Ryan, 2020^36^
**Category 3: Pedagogical improvements**	**Definition**	Reasons pertaining to the desire to improve/introduce a pedagogical aspect (or aspects) of a curriculum
	**Example driver codes**	Increase student‐centred learning, make curricula more integrated, increase experiential/immersion learning, optimising assessment validity/relevance; standardise/improve delivery of learning experiences
	**Example driver statement**	‘At [our institution] we saw the need to revise the curriculum to ensure it was student‐centred’ Fields, 2021^37^
**Category 4: Learner experience/satisfaction**	**Definition**	Reasons pertaining to the desire to improve the learner experience or their satisfaction with a curriculum
	**Example driver codes**	Current curriculum too dense (i.e. volume related); current content too difficult (i.e. complexity related); current content not high yield enough; desire to improve student engagement/enjoyment in the programme
	**Example driver statement**	‘The internal driver was provided by student evaluation, which urged the faculty to introduce electives and opportunity for international exchange’. Kusurkar 2018^38^
**Category 5: Regulatory reasons**	**Definition**	Reasons pertaining to ensuring the curriculum meets relevant mandatory regulatory requirements
	**Example driver codes**	Desire to maintain accreditation/licensing status
	**Example driver statement**	‘Change in our curriculum was driven by the April 2006 joint visit of the Liaison Committee on Medical Education and the Committee for Accreditation of Canadian Medical Schools (LCME/CACMS), which found our school to be non‐compliant on several important educational standards. As a result, the school was placed on probation, and withdrawal of accredited status was threatened’. White 2013^39^
**Category 6: Learner outcomes**	**Definition**	Reasons pertaining to the desire to improve learner outcomes from the currriculum
	**Example driver codes**	Improve preparedness for work; improve preparedness for further/future study
	**Example driver statement**	‘The results of an objective structured clinical examination (OSCE) at one US dental school failed to yield expected outcomes for students' radiographic interpretation skills … The pilot OSCE 2.9% first attempt pass rates initiated curricular revision’. Kumar 2019^40^
**Category 7: Practical/business factors**	**Definition**	Reasons pertaining to the practicalities of delivering the programme or the financial performance/viability of the programme
	**Example driver codes**	Course capacity issues; timetabling/scheduling issues; logistics of clinical placement (e.g. finding hosts); staffing issues (e.g. shortages); financial concerns (e.g. costs of delivering course); grant funding received facilitating curricula change
	**Example driver statement**	‘To help address this increasing student demand with existing faculty and staff capacity, the eCampus Center and the Health Sciences departments collaborated to redesign three bottleneck courses for online delivery’. Chen 2015^41^
**Category 8: Industry benchmarking/reputation factors**	**Definition**	Reasons pertaining to the desire for a programme to meet voluntary industry benchmarks/recommendations, including the desire to be (or be consistent with) an industry exemplar
	**Example driver codes**	Alignment with national recommended curricula; alignment with other externally recommended curricula/guideline; desire to align with industry benchmarks/exemplars; response to national examination change
	**Example driver statement**	‘There has been a global move for change in medical education with key documents coming from the Association of American Medical Colleges, the General Medical Council in the United Kingdom (2009) and from the Irish Medical Council. In response [our institution] introduced a new 5‐year curriculum’ Finn 2014^42^
**Category 9: Community/patient needs**	**Definition**	Reasons pertaining to the desire for a curriculum to be aligned with community/patient needs
	**Example driver codes**	Improve patient outcomes; response to current/future clinical workforce shortages; response to change in profile of community health care needs
	**Example driver statement**	‘The proportion of persons aged 65 and older in Singapore will increase from 9.0% in 2013 to 18.7% in 2030. [Our institution] has responded to the change by significantly increasing curricular time devoted to geriatric medicine teaching to help graduates better meet the health care needs of an aging population’. Koh, 2015^43^
**Category 10: Periodic renewals**	**Definition**	Reasons pertaining to the belief that curricula should be renewed at periodic intervals
	**Example driver codes**	Regular periodic renewal, irregular periodic renewal, renewal triggered by faculty change
	**Example driver statement**	‘Periodic curriculum reform was implemented in both pathology and radiology teaching’ Atta, 2018^44^

Our criteria for creation of a category were a group of related drivers with well demarcated, intuitive boundaries and minimal overlap with other categories. ‘Regulatory reasons’ and ‘industry benchmarking/reputation factors’ were considered two separate categories, with the key difference being that ‘regulatory reasons’ are mandatory and ‘benchmarking/reputation factors’ voluntary. In contrast, a number of ‘joint’ categories were formulated where there was inherently interlinked phenomena that are not easily separated. For example, ‘knowledge/skill issues’ was assigned a single category as knowledge is generally required to perform a skill optimally. Similarly, ‘practical/business factors’ are intertwined (e.g. if there is a practical problem with inadequate staffing, this is also a business issue in terms of the viability of delivering the course and may also be influenced by staff remuneration levels).

### 3.4 Frequency of initiating drivers

**FIGURE 2:**
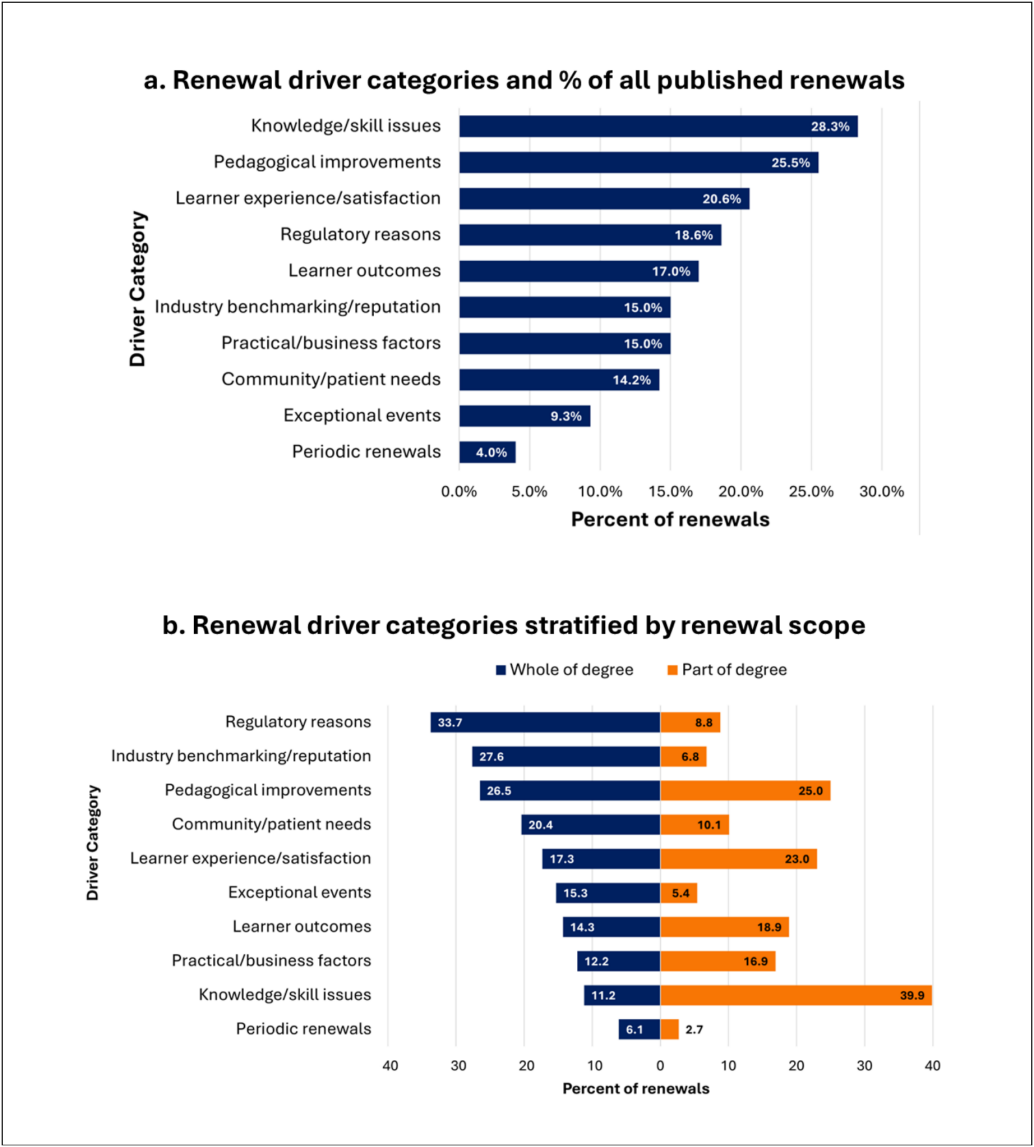
Curriculum renewal driver categories and their frequency. [Color figure can be viewed at wileyonlinelibrary.com]

The proportion of renewals that were initiated by each driver varied widely. When looking across all renewals, ‘knowledge/skill issues’ were the most common driver (70/247 renewals, 28.3%) followed by ‘pedagogical improvements’ (63/247, 25.5%). ‘Periodic renewals’ were the least frequently cited driver (10/247, 4.0%). Figure 2a.

When stratified by the size of the renewal, assymetries arise. Whole‐of‐degree renewals showed a predominance of ‘regulatory’ and ‘industry benchmarking/reputation’ drivers but were rarely initiated for ‘knowledge/skill issues’. A converse pattern was revealed for part‐of‐degree renewals, with knowledge/skill issues dominating and minimal initation for regulatory reasons (Figure 2b).

‘Pedagogical evolutions’, ‘learner experience/satisfaction’ and ‘learner outcomes’ were more equally balanced, being an initiating driver in ~25%, ~20% and ~16% of renewals in both arms, respectively.

## 4 DISCUSSION

This study provides a comprehensive view of real‐life drivers of curriculum renewal in entry‐to‐practice HPE degrees. The creation of a taxonomy of drivers can serve as an important checklist for institutions considering a curriculum renewal to ensure they have considered all potential dimensions of need *prior* to embarking on renewal. It can also help to scaffold future educational research by serving as a map of the different areas of renewal need and thus provide targeted areas of academic inquiry that can be explored individually and in relation to one other.

Curriculum renewal is essential to keep HPE programmes in line with best practice and, thus, fit‐for‐purpose. Recognition of this core role has resulted in a growing body of work looking at understanding HPE curricular renewal processes and their effective implementation.^20^, ^23^, ^50^, ^51^ While every aspect of the process is worthy of scrutiny, the initial step—when the decision is made to commence a renewal process—is especially important.^7^, ^16^ It is a trajectory changing moment and, once made, mandates the mobilisation of significant human and financial capital^2^, ^7^, ^52^; resources that must be judiciously utilised in all HPE settings.^6^, ^21^ Hence, the reasons ‘driving’ this initial decision were chosen as the focus of this study.

To do this in a data‐driven way, it was important to distinguish between actual ‘drivers’ of curricula renewal and theoretical ‘rationales’ for curricula renewal. This difference was best explained by Sklar who once compared curriculum renewal to buying a new car^16^—to extend this analogy, there are many possible ‘rationales’ to buy a new car (increased safety ratings, more space), but the actual ‘driving’ reason may be a mechanic declaring your current car unroadworthy (i.e. an accreditation review). Thus, we specifically examined *drivers* of curricula renewal as explicitly stated by the institutions themselves rather than rationales, which may or may not represent actual practice.

Unsurprisingly, the range of drivers was found to be extremely wide—77 drivers were identified in total that could be grouped into 10 overarching categories. Although none of these categories is completely new to the literature, to our knowledge, they have not been comprehensively compiled and categorised from data until this moment. In the only analogous process we are aware of, Christakis looked at recommended reasons for renewing US medical curricula in 1995,^26^ and all eight drivers which he reported can be reclassified into four of our generated categories—providing a form of validation for the comprehensiveness of our dataset.

When designing this study, we took a deliberately broad lens to the concept of ‘renewal’, choosing to not constrain ourselves to a specific definition for two reasons. First, because doing so may have inadvertently introduced ‘blind spots’ into the driver list. And second, because the boundary between ‘minor’ incremental curricular change (e.g. that may occur as part of Continuous Quality Improvement [CQI]) and more ‘major’ top‐to‐bottom curricular renewal is not universally agreed upon.^16^, ^53^, ^54^ Hence, we included publications describing curricular change of any size and stratified the results based on the extent of the renewal. Ultimately, we found that the generated categories of drivers were relevant across all renewal sizes, no matter how large or small, meaning this categorisation could be potentially utilised to direct *both* CQI and more major renewal processes.

That said, there were some fascinating differences in the relative frequency of drivers when stratified by renewal extent. ‘Regulatory’ reasons were the most frequent drivers of whole‐of‐degree renewals, confirming its role as a key lever to effect change^55^, ^56^—academics looking to promote change in HPE would be well served to have in‐depth and regular engagement at this level. Conversely, knowledge/skill issues featured as the most frequent driver for part‐of‐degree renewals and featured rarely at the whole‐of‐degree level. This stands to reason, as medical knowledge is constantly evolving and updating this does not generally require changes to the underlying philosophical underpinnings or structure of a programme.

In addition, the less frequently represented drivers are also worthy of consideration. ‘Community/patient needs’ were identified as a driver of a renewal in just 14.2% of all renewals, the third least commonly cited driver category. Given that catering to community needs is (or should be) a core mission for all HPE degrees,^57^, ^58^ a more prominent role in curriculum renewal may well be warranted. Another poorly cited category was ‘periodic renewals’. This may be in part due to publication or recall bias as the phenomenon of commencing a renewal ‘because we were due’ seems more prominent anecdotally than was found in the literature. Our stance is that periodic review of curricula is important but, following that, curricular renewal should only proceed if a genuine need for change is identified during the review process—an approach consistent with current CQI methodology.^53^, ^59^

The findings of this study raise a number of opportunities for future research. Having formulated this categorisation, a logical next step would be to identify optimal methods that could be used to assess each driver. Our research group is currently pursuing this line of questioning in an attempt to establish objective and validated instruments that can be used to do this. In addition, it would be interesting to interrogate if there were any differences in the importance of the driver categories and whether or not they lead to successful and/or sustained curriculum changes. Finally, our data demonstrated a significant spike in renewals in 2020, potentially due to pedagogical changes mandated by Covid, and this unusual concentration of renewals could be an opportune cohort for further investigation to confirm the drivers and long‐term success of curricular change.

Altogether, the generated renewal categories will serve as a useful checklist for HPE institutions contemplating a curriculum renewal. It will facilitate the comprehensive identification of renewal needs, thus reducing (or avoiding) the oft‐seen scenario where additional unanticipated ‘needs’ are unearthed deep into the renewal process.^21^, ^22^ In addition, it creates a common framework for curriculum scholars to begin disentangling the complex reasons for curriculum renewal. An ideal next step would be the identification/construction of validated instruments assessing each category that could then be used by institutions to evaluate their HPE programmes. This combination of an accepted framework alongside validated instruments would significantly streamline and standardise the process of HPE curricula renewal.

### 4.1 Limitations

This review remains subject to some limitations. Of note, the dataset was constrained to entry‐to‐practice health professional degrees; therefore, the applicability of these findings to post‐entry settings (e.g. specialisation, CPD) may need to be verified before being utilised in these scenarios. In addition, the fact that this review was collated from published data of actual renewals was both a strength and weakness of this study. Publication bias must be considered as it is feasible that there may be other drivers of renewal that are not being disclosed in the literature (that said, we were genuinely surprised by how candid some institutions were in expressing their own shortcomings, perhaps mitigating this issue). For practical reasons, we limited ourselves to English‐speaking literature, introducing the risk of Geographic bias to this study, with non‐English‐speaking regions of the world comparatively underrepresented.

## 5 CONCLUSION

This review itemises initiating drivers of curriculum renewal in HPE degrees as recorded in the academic literature. It proposes 10 categories of driver, generated via inductive CCA, that can be used as a framework for individual curricular renewals and also to inform future scholarly inquiry. In doing so, this study hopes to make an incremental contribution to the way in which health professional educators and scholars approach the concept of curricular renewal.

## AUTHOR CONTRIBUTIONS

**David L Kok**: Conceptualization; methodology; data curation; software; investigation; formal analysis; project administration; visualisation; writing—original draft; writing—review and editing. **Mary Xu**: Methodology; investigation; validation; formal analysis; writing—review and editing. **Stephen Trumble**: Methodology; investigation; validation; formal analysis; writing—review and editing. **Kristie Matthews**: Conceptualization; methodology; investigation; formal analysis; supervision; writing—review and editing. **Caroline Wright**: Conceptualization; methodology; investigation; formal analysis; supervision; writing—review and editing.

## CONFLICT OF INTEREST STATEMENT

The authors declare that there are no conflicts of interest in relation to this work.

## ETHICAL APPROVAL

Ethics approval was not sought as the manuscript does not report on or involve human participants, human data or human tissue.

## Supporting information


**Appendix S1.** Search strategy.

**Appendix S2.** Supplementary Material.

**Appendix S3.** Full list of drivers.

**Figure S1.** Infographic of study characteristics.
**Table S2**. Curriculum Renewal Details.

## Data Availability

The data that support the findings of this study are available from the corresponding author upon reasonable request.

## References

[1] Haden NK , Hendricson WD , Kassebaum DK , et al. Curriculum change in dental education, 2003–09. J Dent Educ. 2010;74(5):539‐557. doi:10.1002/j.0022-0337.2010.74.5.tb04901.x 20446373

[2] Norman GR . The birth and death of curricula. Adv Health Sci Educ. 2017;22(4):797‐801. doi:10.1007/s10459-017-9790-1 29189964

[3] DePaola DP . The revitalization of US dental education. J Dent Educ. 2008;72(S2):28‐42. doi:10.1002/j.0022-0337.2008.72.2_suppl.tb04476.x 18250375

[4] Blood AD , Farnan JM , Fitz‐William W . Curriculum changes and trends 2010‐2020: a focused National Review Using the AAMC curriculum inventory and the LCME annual medical school questionnaire part II. Acad Med. 2020;95(9S):S5‐S14. doi:10.1097/ACM.0000000000003484 33626633

[5] Parsell G . Undergraduate curriculum change in North America: a quiet revolution. Med Educ. 2000;34(12):972‐973. doi:10.1046/j.1365-2923.2000.00844.x 11123556

[6] Frenk J , Chen LC , Cohen JS , et al. Health professionals for a new century: transforming education to strengthen health systems in an interdependent world. The Lancet. 2011;376(9756):1923‐1958. 10.1016/S0140-6736(10)61854-5 21112623

[7] Bland CJ , Starnaman S , Wersal L , Moorhead‐Rosenberg L , Zonia SC , Henry RC . Curricular change in medical schools: how to succeed. Acad Med. 2000;75(6):575‐594. doi:10.1097/00001888-200006000-00006 10875502

[8] McKerrow I , Carney PA , Caretta‐Weyer H , Furnari M , Miller JA . Trends in medical students' stress, physical, and emotional health throughout training. Med Educ Online. 2020;25(1):1709278. doi:10.1080/10872981.2019.1709278 31902315 PMC6968533

[9] Lyndon MP , Henning MA , Alyami H , Krishna S , Yu TC , Hill AG . The impact of a revised curriculum on academic motivation, burnout, and quality of life among medical students. J Med Educ Curric Dev. 2017;4:2382120517721901. doi:10.1177/2382120517721901 29349339 PMC5736290

[10] Arvandi Z , Emami A , Zarghi N , Alavinia SM , Shirazi M , Parikh SV . Linking medical faculty stress/burnout to willingness to implement medical school curriculum change: a preliminary investigation. J Eval Clin Pract. 2016;22(1):86‐92. doi:10.1111/jep.12439 26563562

[11] Farber J , Payton C , Dorney P , Colancecco E . Work‐life balance and professional quality of life among nurse faculty during the COVID‐19 pandemic. J Prof Nurs. 2023;46:92‐101. doi:10.1016/j.profnurs.2023.03.005 37188429 PMC10027548

[12] Nyoni CN , Goddard VCT . Needs of early adopters in supporting a nursing curriculum innovation in a low resource setting: an exploratory case study. Nurse Educ Today. 2021;104:105002. doi:10.1016/j.nedt.2021.105002 34126325

[13] Aziz S , Wajid G , Khan RA , Zaidi FZ . Dimensions of challenges in transformation from traditional to integrated modular curriculum: experiences from Pakistan. Pak J Med Sci. 2023;39:1730‐1736. doi:10.12669/pjms.39.6.6730 37936778 PMC10626084

[14] Field M , Committee on the Future of Dental Education . Dental education at the crossroads: challenges and change. National Academies Press; 1995. 366 p 25121229

[15] Cusano R , Busche K , Coderre S , Woloschuk W , Chadbolt K , McLaughlin K . Weighing the cost of educational inflation in undergraduate medical education. Adv Health Sci Educ. 2017;22(3):789‐796. doi:10.1007/s10459-016-9708-3 27552815

[16] Sklar DP . Implementing curriculum change: choosing strategies, overcoming resistance, and embracing values. Acad Med. 2018;93(10):1417‐1419. doi:10.1097/ACM.0000000000002350 30252731

[17] Wray N , McCall L . ‘They don't know much about us’: educational reform impacts on students' learning in the clinical environment. Adv Health Sci Educ. 2009;14(5):665‐676. doi:10.1007/s10459-008-9146-y 19031000

[18] Whitehead C , Kuper A , Kuper A . Faith‐based medical education. Adv Health Sci Educ. 2017;22(1):1‐3. doi:10.1007/s10459-016-9748-8 28039589

[19] Blaauw D , Ditlopo P , Rispel LC . Nursing education reform in South Africa–lessons from a policy analysis study. Glob Health Action. 2014;7(1):26401. doi:10.3402/gha.v7.26401 25537941 PMC4275647

[20] Bordage G , Harris IB . Making a difference in curriculum reform and decision‐making processes. Med Educ. 2011;45(1):87‐94. doi:10.1111/j.1365-2923.2010.03727.x 21155872

[21] Onyura B , Lass E , Lazor J , Zuccaro L , Hamza DM . Vitalizing the evaluation of curricular implementation: a framework for attending to the “how and whys” of curriculum evolution. Adv Health Sci Educ. 2021;15(2):1‐20. doi:10.1007/s10459-021-10083-6 34779952

[22] Begenau J , Kiessling C . The Berlin reformed curriculum in undergraduate medical education: a retrospective of the development history, principles, and termination. GMS J Med Educ. 2019;36(5):Doc62. 31815172 10.3205/zma001270 PMC6883246

[23] McLeod PJ , Steinert Y . Twelve tips for curriculum renewal. Med Teach. 2015;37(3):232‐238. doi:10.3109/0142159X.2014.932898 25010218

[24] Novak DA , Hallowell R , Ben‐Ari R , Elliott D . A continuum of innovation: curricular renewal strategies in undergraduate medical education, 2010‐2018. Acad Med. 2019;94:S79‐S85. doi:10.1097/ACM.0000000000002909 31365397

[25] Waters C , Rochester S , McMillan MA . Drivers for renewal and reform of contemporary nursing curricula: a blueprint for change. Contemp Nurse. 2012;41(2):206‐215. doi:10.5172/conu.2012.41.2.206 22800387

[26] Christakis NA . The similarity and frequency of proposals to reform US medical education: constant concerns. JAMA. 1995;274(9):706‐711. doi:10.1001/jama.1995.03530090038019 7650823

[27] Marope M . Reconceptualizing and Repositioning Curriculum in the 21st Century: A Global Paradigm Shift. International Bureau of Education, UNESCO, 2017. Available from: https://www.fundacionsantillana.com/wp-content/uploads/2020/04/reconceptualizing_and_repositioning1.pdf

[28] American Council of Education . Drivers of Change in Higher Education. 2024. Available from: https://www.acenet.edu/Pages/Engage/Videos/Series-Drivers-of-Change-in-Higher-Education.aspx

[29] O'Neill G . Initiating curriculum revision: exploring the practices of educational developers. Int J Acad Dev. 2010;15(1):61‐71. doi:10.1080/13601440903529927

[30] Gruba P , Moffat A , Sondergaard H , Zobel J . What drives curriculum change? ACE. 2004;4.

[31] Law M , Veinot P , Mylopoulos M , Bryden P , Brydges R . Applying activity theory to undergraduate medical curriculum reform: lessons in contradictions from multiple stakeholders' perspectives. Med Teach. 2022;24(7):1‐12. doi:10.1080/0142159X.2022.2041190 35199616

[32] Arksey H , O'Malley L . Scoping studies: towards a methodological framework. Int J Soc Res Methodol. 2005;8(1):19‐32. doi:10.1080/1364557032000119616

[33] Levac D , Colquhoun H , O'brien KK . Scoping studies: advancing the methodology. Implement Sci. 2010;5(1):69. doi:10.1186/1748-5908-5-69 20854677 PMC2954944

[34] Pollock D , Peters MD , Khalil H , et al. Recommendations for the extraction, analysis, and presentation of results in scoping reviews. JBI Evid Syn. 2023;21(3):520‐532. 10.11124/JBIES-22-00123 36081365

[35] Weatherford BH , Viveiros JA . Senior nursing students' perspectives on safety competencies: an end‐of‐program outcome evaluation. Nurs Educ Perspect. 2015;36(3):182‐184. doi:10.5480/13-1182

[36] Ryan TJ , Sheachnasaigh EN , Ryder SA . Design and implementation of online patient‐facing experiences for an integrated pharmacy programme. Pharm Educ. 2020;20(2):160‐164. doi:10.46542/pe.2020.202.160164

[37] Fields L , Trostian B , Moroney T , Dean BA . Active learning pedagogy transformation: a whole‐of‐school approach to person‐centred teaching and nursing graduates. Nurse Educ Pract. 2021;53:103051. doi:10.1016/j.nepr.2021.103051 33865084

[38] Kusurkar RA , Daelmans HE , Horrevoets A , de Haan M , van der Meijde M , Croiset G . Reforms in VUmc School of Medical Sciences Amsterdam: student engagement, a minor elective semester and stakeholder collaboration in improving the quality of assessments. Med Teach. 2018;40(5):501‐505. doi:10.1080/0142159X.2018.1445833 29513053

[39] White J , Paslawski T , Kearney R . ‘Discovery learning’: an account of rapid curriculum change in response to accreditation. Med Teach. 2013;35(7):e1319‐e1326. doi:10.3109/0142159X.2013.770133 23444887

[40] Kumar V , Gadbury‐Amyot CC . Predoctoral curricular revision for dental radiographic interpretation competence based on OSCE results. J Dent Educ. 2019;83(10):1233‐1239. doi:10.21815/JDE.019.112 31182621

[41] Chen KZ , Anderson J , Hannah EL , Bauer C , Provant‐Robishaw C . Resolving bottlenecks: converting three high‐enrollment nursing courses to an online format. J Nurs Educ. 2015;54(7):404‐408. doi:10.3928/01484834-20150617-09 26155034

[42] Finn Y , Avalos G , Dunne F . Positive changes in the medical educational environment following introduction of a new systems‐based curriculum: DREEM or reality? Curricular change and the environment. Ir J Med Sci. 2014;183(2):253‐258. doi:10.1007/s11845-013-1000-4 23943152

[43] Koh GC , Ling CL , Ma BH , et al. Effect of a new longitudinal interprofessional geriatric medicine educational track on knowledge and attitude of medical students: a controlled cohort study. J Am Geriatr Soc. 2015;63(3):558‐564. doi:10.1111/jgs.13295 25732398

[44] Atta IS , AlQahtani FN . Integrated pathology and radiology learning for a musculoskeletal system module: an example of interdisciplinary integrated form. Adv Med Educ Pract. 2018;9:527‐533. doi:10.2147/AMEP.S167692 30050333 PMC6055894

[45] Hamilton AL , Layden EA , Storrar N , Skinner J , Harden J , Wood M . Definition, measurement, precursors, and outcomes of trust within health care teams: a scoping review. Acad Med. 2024 Jan;99(1):106‐117. doi:10.1097/ACM.0000000000005320 37433205

[46] Allen LM , Palermo C , Armstrong EG , Hay M . Categorising the broad impacts of continuing professional development: a scoping review. Med Educ. 2019;53(11):1087‐1099. doi:10.1111/medu.13922 31396999

[47] Harris PA , Taylor R , Thielke R , Payne J , Gonzalez N , Conde JG . Research electronic data capture (REDCap)—a metadata‐driven methodology and workflow process for providing translational research informatics support. J Biomed Inform. 2009;42(2):377‐381. doi:10.1016/j.jbi.2008.08.010 18929686 PMC2700030

[48] Elo S , Kyngäs H . The qualitative content analysis process. J Adv Nurs. 2008;62(1):107‐115. doi:10.1111/j.1365-2648.2007.04569.x 18352969

[49] Hsieh HF , Shannon SE . Three approaches to qualitative content analysis. Qual Health Res. 2005;15(9):1277‐1288. doi:10.1177/1049732305276687 16204405

[50] Soomro R , Soomro R , Rehman SU , Ali S , McKimm J . Using the ‘twelve tips for applying change models’ for undergraduate medical curriculum reform in Pakistan: incorporating a new trauma evaluation and management TEAM® course. MedEdPublish. 2022;12:29. doi:10.12688/mep.17507.2 36817618 PMC9926506

[51] Davis MH , Harden RM . Planning and implementing an undergraduate medical curriculum: the lessons learned. Med Teach. 2003;25(6):596‐608. doi:10.1080/0142159032000144383 15369907

[52] Deckers PJ . Health care reform and undergraduate medical education: implications for surgeons. Arch Surg. 2000;135(4):399‐408. doi:10.1001/archsurg.135.4.399 10768704

[53] Hedrick JS , Cottrell S , Stark D , et al. A review of continuous quality improvement processes at ten medical schools. Med Sci Educ. 2019;29(1):285‐290. doi:10.1007/s40670-019-00694-5 34457478 PMC8368582

[54] Hubers MD . Paving the way for sustainable educational change: Reconceptualizing what it means to make educational changes that last. Teach Teach Educ. 2020;93:103083. doi:10.1016/j.tate.2020.103083

[55] Cuff PA , Perez MM . Exploring the role of accreditation in enhancing quality and innovation in health professions education: proceedings of a workshop; 2017 Apr 7. doi:10.17226/23636 28609024

[56] Frank JR , Frank JR , Taber S , van Zanten M , Scheele F , Blouin D . The role of accreditation in 21st century health professions education: report of an international consensus group. BMC Med Educ. 2020;20(1):305. doi:10.1186/s12909-020-02121-5 32981519 PMC7520947

[57] Lewkonia RM . The missions of medical schools: the pursuit of health in the service of society. BMC Med Educ. 2001;1(1):4. doi:10.1186/1472-6920-1-4 11696255 PMC59665

[58] Rourke J . Social accountability: a framework for medical schools to improve the health of the populations they serve. Acad Med. 2018;93(8):1120‐1124. doi:10.1097/ACM.0000000000002239 29642103

[59] Barzansky B , Hunt D , Moineau G , et al. Continuous quality improvement in an accreditation system for undergraduate medical education: benefits and challenges. Med Teach. 2015;37(11):1032‐1038. doi:10.3109/0142159X.2015.1031735 25897708

