# Supplementary material for: Categorising drivers of curriculum renewal in entry‐to‐practice health professional education: A scoping review
Source: Med Educ. 2025 Feb 19;59(8):812–22. doi: 10.1111/medu.15614 (PMC12242900; doi:10.1111/medu.15614)
Supplement: Supplementary file 1 — Appendix S1. Search strategy. [file MEDU-59-812-s004.docx]

# Appendix S1 – Search strategy

| Concept 1 (all health professions) | Concept 2 (entry to practice qualification ) | Concept 3 (curriculum renewal) |
| --- | --- | --- |
| medic*  or health  or radi*  or "occupational therap*"  or "social work"  or podiatr*  or "exercise phys*"  or physiotherap*  or nurs*  or "allied health"  or diet*  or dentist*  or psychology*  or pharmac*  or "speech therap*"  or "speech path*"  or "physical therap* | Degree  or undergraduate  or (medic* or vet* or dent* or nurs* or pharmac* or physioth* or podiatry*) adj school | (curricul* or program or programme or course)  adj1 (new or renew* or reform* or refresh* or reorgani* or redesign* or restructur* or chang* or transfor* or revis*) |
