# Supplementary material for: Categorising drivers of curriculum renewal in entry‐to‐practice health professional education: A scoping review
Source: Med Educ. 2025 Feb 19;59(8):812–22. doi: 10.1111/medu.15614 (PMC12242900; doi:10.1111/medu.15614)
Supplement: Supplementary file 3 — Appendix S3. Full list of drivers. [file MEDU-59-812-s001.docx]

# Appendix S3 – Full list of drivers

| **Category** | **Individual Driver** | **n** |
| --- | --- | --- |
| Pedagogical Improvements | Increase PBL/active learning | 8 |
|  | Increase self-directed learning | 1 |
|  | Increase team-based learning | 4 |
|  | Increase student-centred learning | 10 |
|  | Increase Interprofessional/Multidisciplinary Education | 7 |
|  | Make curricula more integrated | 13 |
|  | Increase system-based learning | 1 |
|  | Increase authentic experiential/immersion learning | 8 |
|  | Increase patient-centred learning | 3 |
|  | Increase reflective practice | 4 |
|  | Increase competency-based learning | 2 |
|  | Increase curricula cohesiveness/alignment | 8 |
|  | Increase mentoring/apprenticeship | 4 |
|  | Increase flipped-classroom learning | 1 |
|  | Increase online learning/e-learning | 1 |
|  | Optimizing assessment validity/relevance | 8 |
|  | Standardize/improve delivery of learning experiences | 4 |
|  | Direct statement about desire for pedagogical improvement | 12 |
|  | Improve teacher-student ratios | 1 |
| Knowledge/skill Issue | Topic underrepresented | 64 |
|  | Topic requires updating to be more contemporary (including removal of outdated knowledge) | 5 |
|  | Topic overrepresented | 1 |
| Learner experience/satisfaction factors | Current curriculum too dense (ie. Volume related) | 14 |
|  | Current curriculum too difficult (i.e. Complexity related) | 6 |
|  | Current content not high yield enough | 8 |
|  | Desire to increase continuity/cohesiveness of curriculum | 9 |
|  | Desire to increase flexibility of curriculum | 5 |
|  | Desire to increase structure in the curriculum | 1 |
|  | Desire to improve student engagement/enjoyment in the program | 10 |
|  | Desire to reduce anxiety/stress of students | 5 |
|  | Desire to reduce internal competition between students | 3 |
|  | Direct statement about learner experience/satisfaction | 17 |
|  | Desire to improve uniformity of learner experience | 2 |
|  | Desire to reduce emotional distress of students | 1 |
|  | Desire to reduce student fear of failure | 1 |
| Learner outcomes | Improve knowledge retention | 1 |
|  | Improve preparedness for work | 21 |
|  | Improve preparedness for further/future study | 6 |
|  | Improve examination performance | 8 |
|  | Direct statement about improving learner outcomes | 12 |
| Practical/business factors | Course capacity issues | 6 |
|  | Desire to reduce total program duration | 4 |
|  | Timetabling/scheduling issues | 6 |
|  | Physical location issues | 2 |
|  | Issues with the logistics of clinical placement (e.g. finding adequate hosts) | 5 |
|  | Desire to improve staff workload/conditions | 3 |
|  | Staffing issues (e.g. shortages) | 4 |
|  | Financial concerns (e.g. Costs of delivering course) | 2 |
|  | Desire to increase enrolments | 2 |
|  | Incentive was provided for curricula change | 4 |
|  | Received grant funding facilitating curricula change | 14 |
|  | Direct statement about Practical/Business factors | 3 |
|  | Change in university macro structure | 1 |
|  | Improve quality control across multiple sites | 1 |
|  | Establishment of new campus/centre | 1 |
| Regulatory reasons | Desire to maintain accreditation/licensing status | 45 |
|  | Direct statement about Regulatory reasons | 1 |
| Exceptional events | Covid-19 | 22 |
|  | War | 1 |
| Community/patient needs | Improve patient outcomes | 5 |
|  | Response to current/future clinical workforce shortages | 11 |
|  | Response to current/future research workforce shortages | 2 |
|  | Response to change in workforce profile (eg. more casualisation/gender) | 2 |
|  | Response to change in profile of community healthcare needs | 14 |
|  | Direct statement about Community/Patient factors | 11 |
| Industry benchmarking/reputation | Response to national examination change | 2 |
|  | Alignment with national recommended curricula | 11 |
|  | Alignment with other externally recommended curricula/guideline | 5 |
|  | Desire to be seen as an industry benchmark/exemplar | 3 |
|  | Desire to align with industry benchmarks/exemplars | 7 |
|  | Desire to meet baseline industry expectations | 7 |
|  | Related to involvement in an educational partnership/alliance | 6 |
|  | Direct statement about Industry benchmarking/reputation | 3 |
|  | Other industry benchmarks/recommendations | 2 |
| Periodic renewal | Regular Periodic Renewal | 5 |
|  | Irregular Periodic Renewal | 4 |
|  | Renewal triggered by faculty change (e.g. New Dean) | 1 |
| **Total** |  | **532** |
