# Supplementary material for: Categorising drivers of curriculum renewal in entry‐to‐practice health professional education: A scoping review
Source: Med Educ. 2025 Feb 19;59(8):812–22. doi: 10.1111/medu.15614 (PMC12242900; doi:10.1111/medu.15614)
Supplement: Supplementary file 4 — Figure S1. Infographic of study characteristics. Table S2. Curriculum Renewal Details. [file MEDU-59-812-s003.docx]

# Supplementary Materials


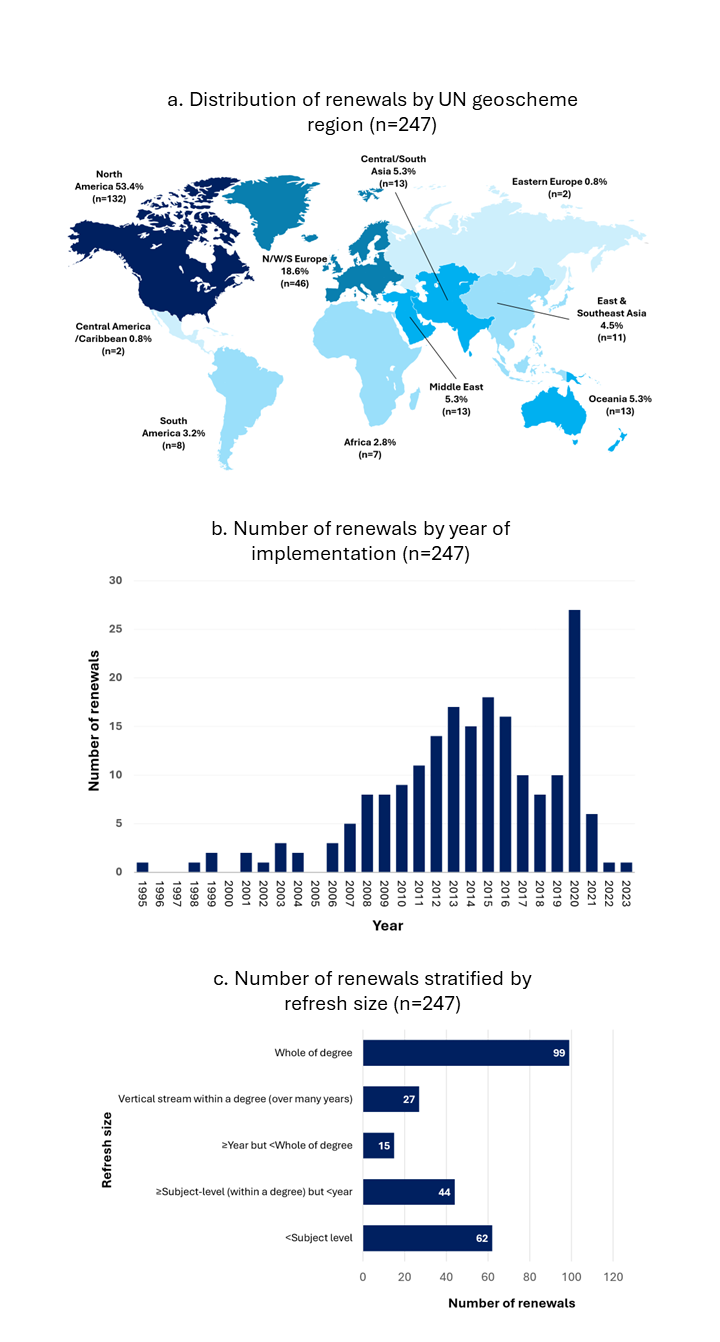


**Supplementary Figure S1 – Infographic of study characteristics**

**Supplementary Table S2 – Curriculum Renewal Details**

| Whole of degree  Renewal Specifics (more than one may apply) | No.  (n=99) | % |  | Part of degree Renewal Specifics (more than one may apply) | No.  (n=148) | % |
| --- | --- | --- | --- | --- | --- | --- |
| New/changed pedagogy | 79 | 79.8% |  | New/changed pedagogy | 63 | 42.6% |
| Move to online (either full or partial) | 17 | 17.2% |  | New subject/elective/vertical stream in degree | 28 | 18.9% |
| Unable to classify | 3 | 3.0% |  | New/changed topic/subject matter included | 90 | 60.8% |
|  |  |  |  | New/changed assessment | 11 | 7.4% |
|  |  |  |  | Move to fully online | 6 | 4.1% |
|  |  |  |  | Move to hybrid | 6 | 4.1% |
|  |  |  |  | Move to f2f | 0 | 0.0% |
|  |  |  |  | None of the above/not stated | 13 | 8.8% |
